# A comparative study of thumb reconstruction through the transplant of the first toe compound free flap between emergency surgery and elective surgery

**DOI:** 10.1097/MD.0000000000030196

**Published:** 2022-08-26

**Authors:** Lei Ge, Qiandong Liu, Xiangyun Wang, Qiang He, Lei Zhang, Libin Lu, Qinglin Dong, Yang Gao

**Affiliations:** a Department of Emergency, People’s Hospital of Rizhao, Jining Medical University, Shandong, China; b Department of Medical Instruments, People’s Hospital of Rizhao, Jining Medical University, Shandong, China.

**Keywords:** elective surgery, emergency surgery, thumb reconstruction

## Abstract

This study compared emergency surgery with elective surgery for thumb reconstruction to explore the advantages, safety, and clinical value of emergency reconstruction. By comparing the advantages and disadvantages of thumb reconstruction in emergency surgery and elective surgery, it provides data support for optimizing the treatment process and methods. In this study, 22 patients who underwent thumb reconstruction in Rizhao people’s Hospital from January 2018 to December 2020 were randomly divided into emergency operation group and elective operation group. The differences in operation period, hospitalization time, postoperative complications, hand function score, and satisfaction score between the 2 groups were analyzed. The operation period and hospitalization time of patients in the emergency surgery group were significantly lower than those in the elective surgery group, with statistical significance (*P* < .05). There was no significant difference in postoperative complications between the 2 groups (*P* > .05). After 3 months of rehabilitation training, the 2-point discrimination, functional score, and satisfaction score of the reconstructed thumb in the emergency surgery group were higher than those in the elective surgery group, and the difference was statistically significant (*P* < .05). Emergency reconstruction of the thumb can reduce operation time and hospitalization time, reduce operation costs, and obtain a more ideal appearance and function.

## 1. Introduction

The thumb plays a vital role in the function of the hand and is an indispensable part of gripping, pinching, and other actions. The shape and length of the thumb are the key factors affecting the function of the thumb. From a functional point of view, the thumb accounts for about 40% of the function of the entire hand, especially the grip function which accounts for 80% of the function of the hand, which is inseparable from the cooperation of the thumb.^[1]^ From the perspective of aesthetics, hand trauma, especially thumb amputation, is not conducive to mental health, and it will lead to inferiority complex and depression.^[2]^ The most common type of thumb amputation is the amputation far from the interphalangeal joint, and the repair and reconstruction of this part of the thumb defect can bring significant improvement in function and appearance to patients.^[3]^ Mid-distal defect of the thumb is the most common type (fingertip to metacarpal neck). Distal third reconstruction usually requires only soft tissue restoration.^[4]^ Many options exist for middle third reconstruction, including increasing thumb ray length (metacarpal lengthening, osteoplastic reconstruction, toe transfer) and increasing relative length (phalangization).^[4]^ But these surgical options are not satisfactory from a cosmetic point of view. We recreated the thumb through the transplant of the first toe compound free flap. The reconstructed thumb has a natural appearance, and all the toes are preserved.

For thumb amputation, because it is in an abnormal state after injury, if it is not treated in time, it will inevitably lead to tendon and muscle atrophy, which will bring great difficulties to thumb reconstruction and functional exercise.^[5]^ Therefore, how to choose the best operation time for thumb reconstruction is the focus and difficulty in clinical work. The purpose of this study is to provide data support for the timing of thumb reconstruction by comparing the advantages and disadvantages of emergency surgery and elective surgery.

## 2. Materials and Methods

### 2.1. General data

This study was carried out on 22 patients with thumb amputation, admitted to Rizhao People’s Hospital from January 2018 to December 2020, including 13 male patients and 9 female patients. Patients were randomly divided into the emergency operation group and the elective operation group by randomized double-blind method, with 11 patients in each group. The study was approved by the ethical committees of the participating hospital. All patients signed informed consent. All clinical investigations were conducted according to the principles expressed in the declaration of Helsinki.

### 2.2. Inclusion and exclusion criteria

#### 2.2.1. Inclusion criteria.

The degree of thumb loss is mid and distal (tip to metacarpal neck); patients and their families willing to reconstruct the thumb; no other serious diseases and can tolerate surgery.

#### 2.2.2. Exclusion criteria.

Patients with systemic diseases who are not suitable for thumb reconstruction; patients with chronic diseases and poor general condition; history of trauma in the foot donor site; patients with varicose veins on the feet and fungal bacterial infection; patients with poor compliance.

### 2.3. Operative procedure

#### 2.3.1. Emergency surgery group.

The thumb was reconstructed with a toenail, bone, and skin composite tissue flap within 6 hours after injury; elective surgery group: patients underwent debridement in the emergency room, and all patients were admitted to the hospital for antibiotics to prevent infection, and dressings were changed regularly. The patient underwent thumb reconstruction after the wound was free of infection.

The second toe transplantation to reconstruct the thumb is the most important method of finger reconstruction in the past. But it is not satisfactory from an appearance point of view. We recreated the thumb through the transplant of the first toe compound free flap. The reconstructed thumb has a natural appearance, and all the toes are preserved.

**Figure 1.**
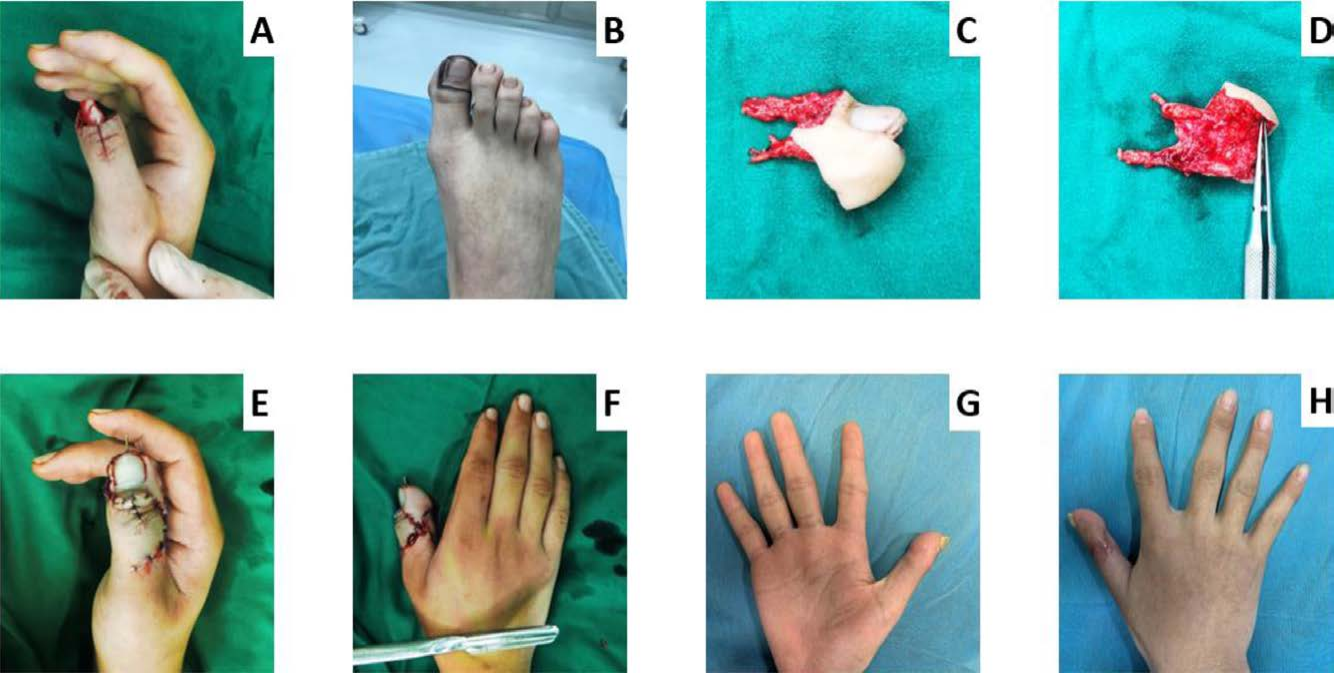
Surgical steps for thumb reconstruction with toenail, bone, and skin composite tissue flaps. (A) Patients with amputation beyond the interphalangeal joint of the thumb. (B) Designing flaps on the foot. (C, D) According to the defect of the thumb, a composite tissue flap of toenail, bone and skin was dissect from the foot. (E, F) The defect was covered and reconstructed with the toenail, bone, and skin composite tissue flaps. (G, H) Three months after surgery satisfactory aesthetic results.

Surgical steps for thumb reconstruction with toenail, bone, and skin composite tissue flaps (Fig. 1). According to the condition of the thumb defect, an experienced doctor dissect a toenail, bone, and skin composite tissue flap from the foot, with the artery as the plantar artery, the vein as the dorsal vein and the nerve as the plantar nerve. Then, the excised toenail, bone, and skin composite tissue flaps were trimmed. The flaps were rolled up to wrap the phalanges, assembled to the shape of the original finger defect, and then transplanted to the thumb stump to reconstruct the thumb like a severed finger replantation (plantar artery and digital artery anastomosis, plantar nerve, and digital nerve anastomosis, dorsal toe vein and digital dorsal vein anastomosis). The donor site of the foot is directly sutured or repaired with free skin grafting.

The patient stayed in bed for 1 week after the operation and provided warmth to his hands through light. Antithrombotic, antibiotics, and antispastic treatment were given, and the blood flow in the hand of the patient was closely monitored. The sutures were removed 2 weeks after the operation, and the Kirschner wires were removed 5 weeks after the operation. Patients perform functional exercises with the help of a professional rehabilitation therapist.

### 2.4. Observation indicators

The gender, age, obesity (BMI > 23), smoking history, red blood cell count (RBC), hemoglobin (HB), total serum protein (TP), serum albumin (ALB), blood glucose (GLU), and glycated serum protein (GSP) were compared between the 2 groups of patients. The operation period and the hospitalization time were compared between the 2 groups. The postoperative complications of the 2 groups were compared. Postoperative complications include swelling, infection, arterial and venous crisis, and skin necrosis. Three months after surgery, the 2-point of discrimination of the reconstructed thumb was compared between the 2 groups. Three months after the operation, the reconstructed thumb function scores of the 2 groups were compared. The comparison criteria were as follows: Score 5—Reconstructed thumb moves normally without effort; Score 3–4—Finger moves normally but with mild pain; Score 2—Finger can barely move with pain; Score 1—Finger does not move. The higher the score, the better the functional recovery. Satisfaction score of patients with reconstructed thumb shape. The comparison criteria were as follows: 10 points—very satisfied, willing to show the reconstructed thumb with others; 7 points—somewhat satisfied, basically willing to show the reconstructed thumb with others; 3 points—average satisfaction, unwilling to show the reconstructed thumb with others; 0 points—dissatisfied with the appearance, refuse to show the reconstructed thumb.

### 2.5. Statistical methods

SPSS18.0 software was used to analyze these data. Data description was expressed as mean ± standard deviation (x ± s), T-test was used for pairwise comparisons, and ANOVA test was used for multiple variables. Test *P* < .05 indicates statistical significance.

## 3. Results

### 3.1. Comparison of general data

We compared the basic information of the 2 groups of patients and found that there was no significant difference between the 2 groups in gender, age, obesity (BMI > 23), smoking history, red blood cell count, hemoglobin, total serum protein, serum albumin, blood glucose, and glycated serum protein (Table 1, *P* > .05).

**Table 1:** Comparison of general data.

	Emergency surgery group	Elective operation group	X^2^	*P*
Gender			0.737	.391
Male	7	6		
Female	4	5		
BMI (kg/m^2^)			0.002	.964
≤23	6	7		
>23	5	4		
Smoking history				
Yes	3	4	0.209	.647
No	8	7		
Age (year)	39.73 ± 3.21	41.73 ± 3.09	0.449	.659
RBC (10^12^/L)	4.612 ± 0.45	4.636 ± 0.21	0.165	.871
Hb (g/L)	139.55 ± 4.33	144.09 ± 7.22	0.938	.363
TP (g/L)	57.47 ± 10.36	62.31 ± 6.25	1.326	.199
ALB (g/L)	35.85 ± 7.03	38.26 ± 4.03	0.986	.336
GLU (mmol/L)	5.36 ± 0.87	5.80 ± 2.16	0.628	.541
GSP (mmol/L)	1.46 ± 0.42	1.96 ± 1.24	1.269	.228

There was no significant difference between the 2 groups in gender, age, obesity (BMI > 23), smoking history, red blood cell count, hemoglobin, total serum protein, serum albumin, blood glucose, and glycated serum protein.

ALB = serum albumin, BMI = body mass index, GLU = blood glucose, GSP = glycated serum protein, HB = hemoglobin, RBC = red blood cell count, TP = total serum protein.

### 3.2. Comparison of the operative period and hospitalization time

The operation period of patients in the emergency operation group was 322.91 ± 16.78, and the operation period of patients in the elective operation group was 384.91 ± 11.86. There was a significant difference with statistical significance (*P* < .05). The hospitalization time of patients in the emergency surgery group was 12.18 ± 1.66, and the hospitalization time of patients in the elective surgery group was 18.09 ± 2.63. There was a significant difference, with statistical significance (Table 2, *P* < .05).

**Table 2:** Comparison of the operative period and hospitalization time.

	Emergency surgery group	Elective operation group	X^2^	*P*
Operative period (min)	322.91 ± 16.78	384.91 ± 11.86	10.005	<.0001
Hospitalization time (d)	12.18 ± 1.66	18.09 ± 2.63	6.307	<.0001

The operative period and hospitalization time of the patients in the emergency surgery group were significantly lower than those in the elective surgery group, and the difference was statistically significant.

### 3.3. Complications in the 2 groups of patients

Swelling occurred in 2 patients in the emergency surgery group and 3 patients in the elective surgery group, which was significantly improved after raising the affected limb and taking Aescuven forte orally. One case of infection occurred in both the emergency surgery group and the elective surgery group, which improved after antibiotic treatment. There were no arterial and venous crises in either the emergency surgery group or the elective surgery group. There was 1 case of partial skin necrosis in the emergency operation group, and 2 cases of partial skin necrosis in the elective operation group, which recovered after dressing change. The sutures were removed 2 weeks after surgery in both groups. There was no significant difference in each index, and there was no statistical significance (Table 3, *P* > .05).

**Table 3:** Complications in the 2 groups of patients.

	Emergency surgery group	Elective operation group	X^2^	*P*
Swelling	2	3	0.259	.611
Infection	1	1		
Arterial and venous crisis	0	0		
Skin necrosis	1	2	0.386	.534

Postoperative complications (swelling, infection, skin necrosis, arterial and venous crises) were not significantly different between the 2 groups and were not statistically significant.

### 3.4. Comparison of 2-point discrimination

**Figure 2.**
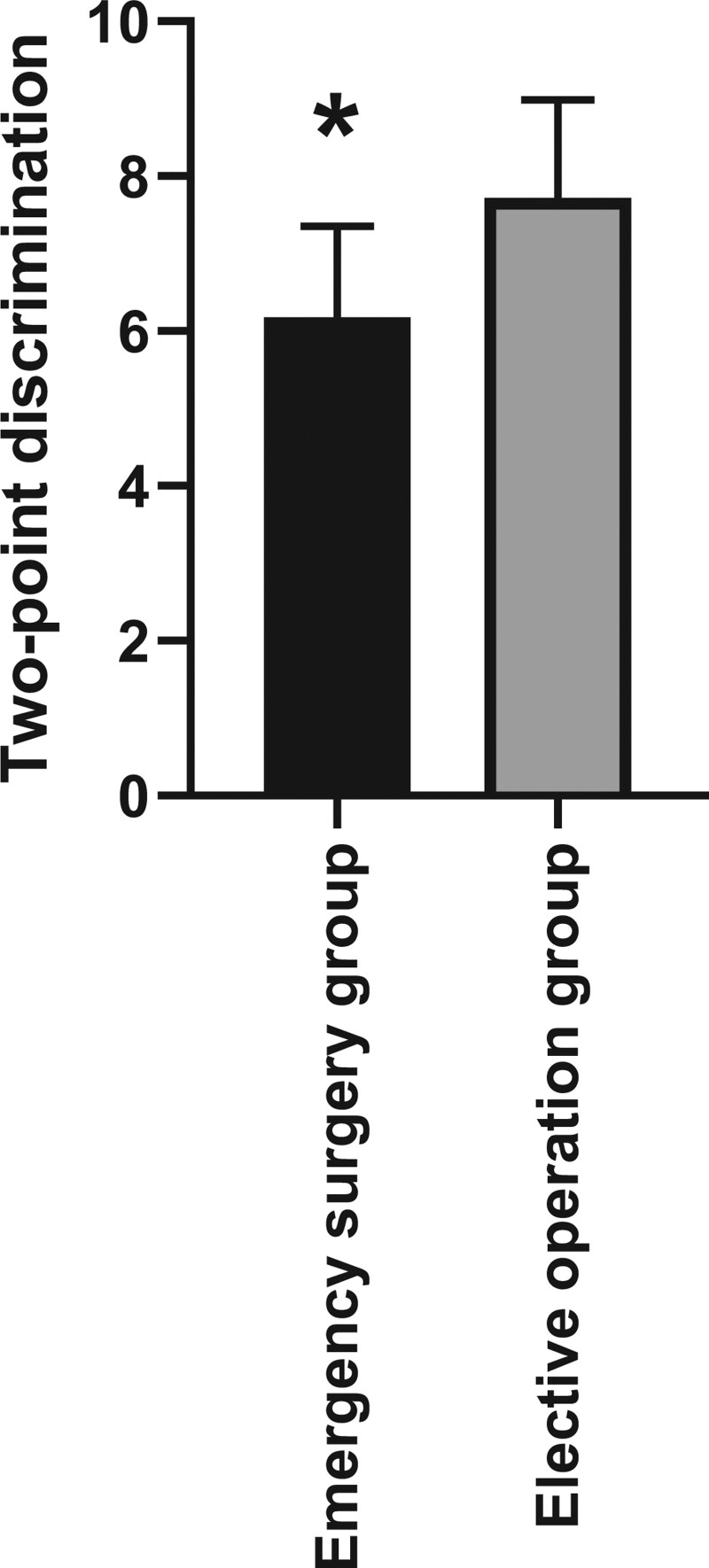
Comparison of 2-point discrimination. After 3 months of rehabilitation training, the 2-point discrimination of the reconstructed thumb in the emergency surgery group was higher than those in the elective surgery group, and the difference was statistically significant.

All patients were treated with oral mecobalamin tablets for 3 months, and the 2-point discrimination of the reconstructed thumb of the 2 groups was measured. The 2-point discrimination of patients in the emergency surgery group was 6.18 ± 1.17, and the 2-point discrimination of patients in the elective surgery group was 7.72 ± 1.27. There was a significant difference between the 2 groups, with statistical significance (figure 2, *P* < .05).

### 3.5. Functional score of reconstructed thumbs

**Figure 3.**
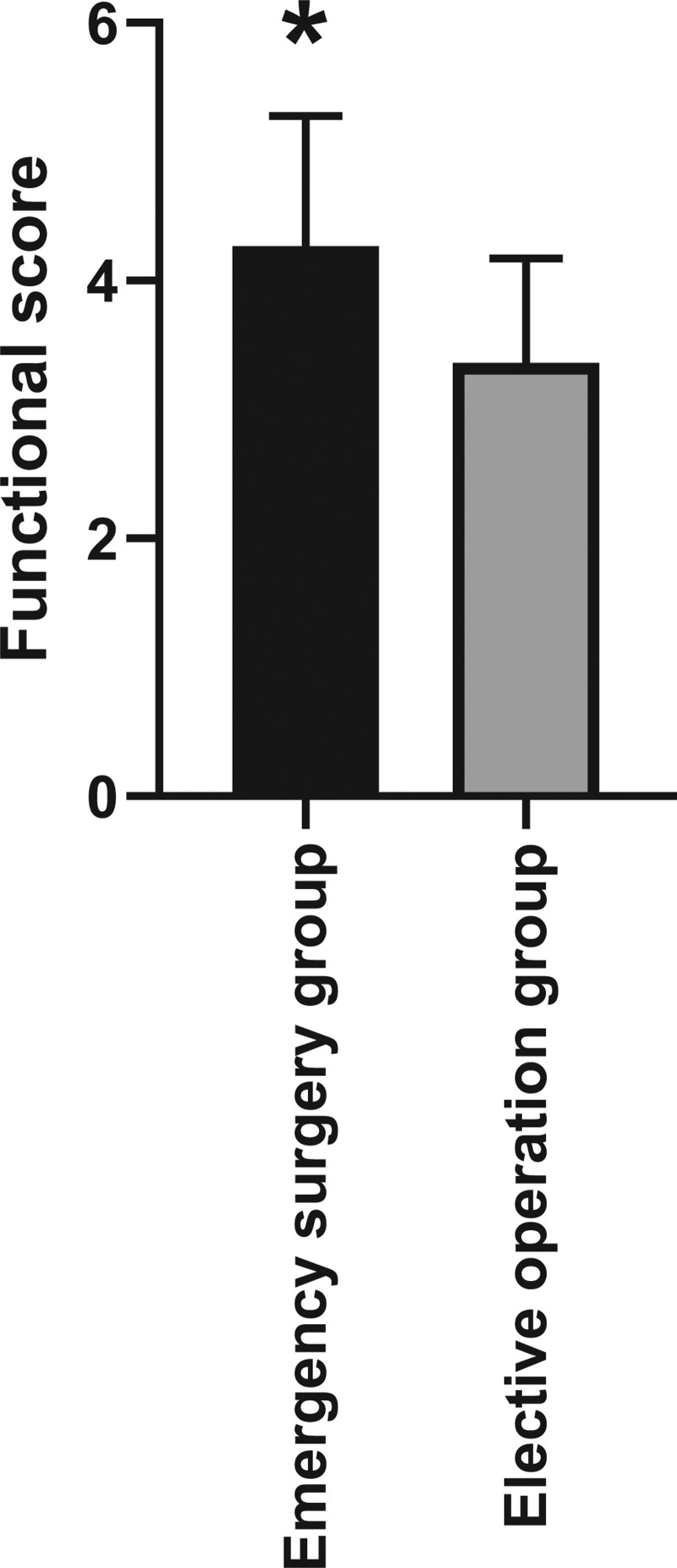
Functional score of reconstructed thumbs. After 3 months of rehabilitation training, the functional score of the reconstructed thumb in the emergency surgery group was higher than those in the elective surgery group, and the difference was statistically significant.

After 3 months of rehabilitation exercises, all patients were evaluated for the function of the reconstructed thumb. The functional score of the patients in the emergency surgery group was 4.27 ± 1.01, and the functional score of the patients in the elective surgery group was 3.36 ± 0.81. There was a significant difference between the 2 groups, with statistical significance (Figure 3, *P* < .05).

### 3.6. Satisfaction score of reconstructed thumbs

**Figure 4.**
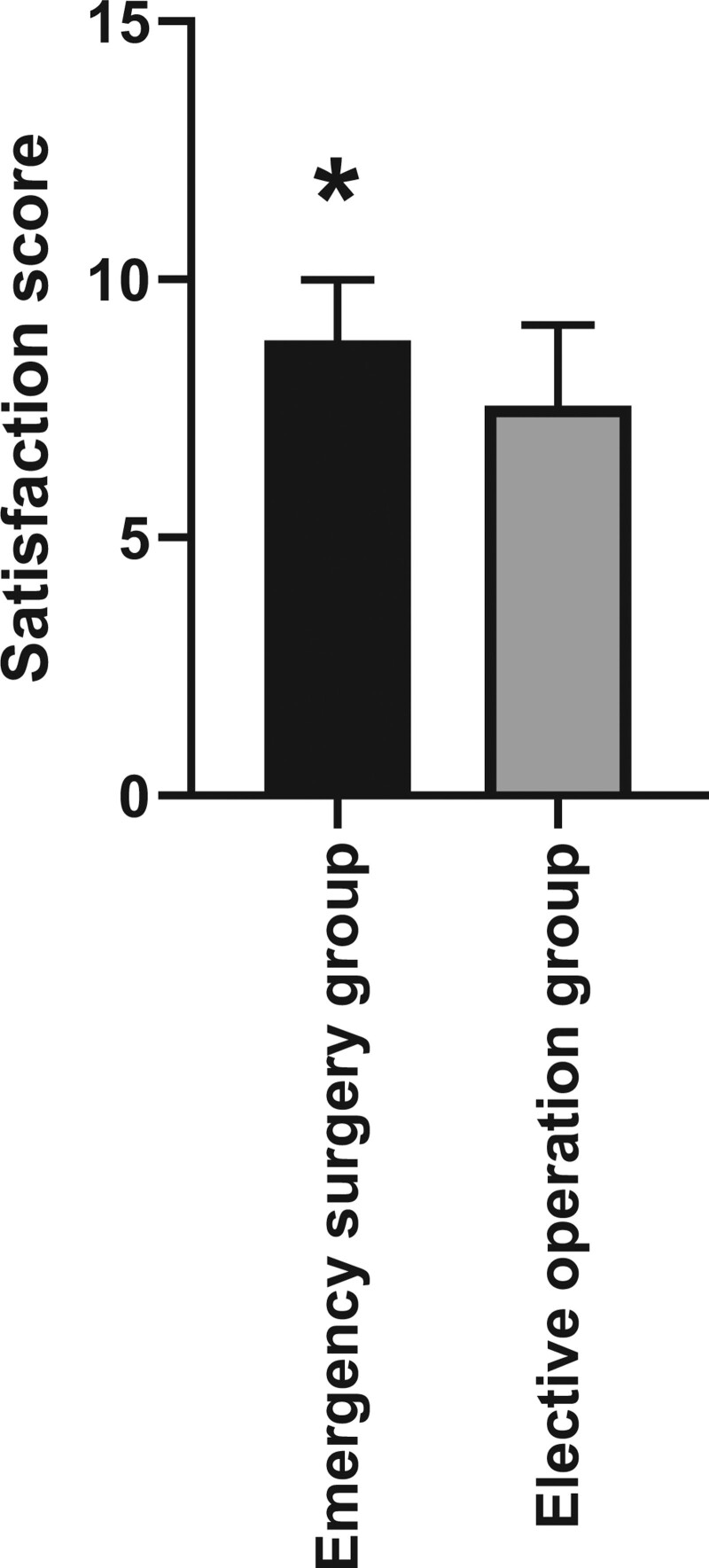
Satisfaction score of reconstructed thumbs. After 3 months of rehabilitation training, the satisfaction score of the reconstructed thumb in the emergency surgery group was higher than those in the elective surgery group, and the difference was statistically significant.

After 3 months of rehabilitation exercises, the patients’ satisfaction scores for reconstructed thumb were counted. The satisfaction score of patients in the emergency surgery group was 8.82 ± 1.17, and the satisfaction score of patients in the elective surgery group was 7.55 ± 1.57. There was a significant difference between the 2 groups, with statistical significance (Fig. 4, *P* < .05).

## 4. Discussion

The thumb accounts for 40% of the function of the hand, and the thumb is the only finger that can oppose the other 4 fingers.^[6]^ If it is missing, the hand will lose nearly half of its function. Thumb amputation not only affects life and work, but also is not conducive to mental health. If not treated in time, it will inevitably cause tendon and muscle atrophy, which will bring great difficulties to thumb reconstruction and functional exercise.^[5]^ Dogu study found that hand trauma is prone to psychological depression in the early stage, which brings great suffering to the recovery of hand function.^[7]^ Therefore, after the thumb is severed, how to rebuild a complete thumb and maximizing its function recovery is an important purpose of hand surgery.

Thumb reconstruction aims to restore basic thumb characteristics and movements, including mobility, stability, sensitivity, length, and appearance.^[8]^ The second toe transplantation to reconstruct the thumb is the most important method of finger reconstruction in the past.^[9,10]^ But it is not satisfactory from an appearance point of view. We reconstruct the thumb with the toenail, bone, and skin composite tissue flaps, disassemble the parts of the toe, and then assemble them according to the needs of the fingers. This reconstructed thumb is the natural appearance, and all the toes are preserved. Mid-distal defects of the thumb are the most common type. The reconstruction of the mid-distal defect of the thumb does not require joint transplantation, the operation time is relatively short, and the risk is small. Therefore, we selected patients with the mid-distal defect of the thumb as the research object. Twenty-two patients included in this study were randomly divided into emergency surgery group and elective surgery group for thumb reconstruction. By comparing the incidence of postoperative complications in the 2 groups, we found that emergency surgery had no obvious advantage in treatment effect compared with elective surgery. With the continuous development of microsurgical technology and the increasing maturity of microvascular anastomosis technology, doctors can achieve the best functional and aesthetic reconstruction through microsurgical technology, and minimize the occurrence of complications.^[11]^

Compared with the patients in the emergency operation group, there was no significant difference in postoperative complications between the 2 groups, but early surgery meant less soft tissue damage and earlier functional exercise, shorter hospital stay, and less cost. In addition, emergency surgery can make it easier to free blood vessels and improve the effect of anastomosis, thereby reducing the operation time and difficulty of the operation. Minhas compared emergency surgery and elective surgery for open fractures of the hand and found that emergency surgery did not increase the infection rate of patients.^[12]^ Georgescu found that emergency toe-to-hand transfer in 31 patients was helpful in reducing surgical trauma.^[13]^ Woo research has shown that in acute hand injuries, immediate toe-to-hand transfer has many advantages, including single-stage reconstruction, shortened recovery period, early return to work, and improved socioeconomic benefits.^[14]^

By comparing the patients in the elective surgery group and the emergency surgery group, we found that the 2-point discrimination, the reconstructed thumb function score, and the satisfaction score in the emergency surgery group were better than those in the elective surgery group. This may have the following 3 reasons: First, more important tissues such as tendons, bones, and joints can be retained during emergency reconstruction, which can maximize the recovery of the mobile function of the thumb. Second, emergency surgery can preserve longer nerve stumps, retain more sensory innervation planes, and reduce the chance of nerve tissue entrapment and adhesion between surrounding scar tissue, so the sensory recovery is better. Third, compared with elective surgery, emergency surgery reduces 1 debridement process and causes less trauma to the hand, which can effectively reduce scar formation and avoid nail deformity, so patient satisfaction is higher. Fourth, patients who underwent elective surgery were more likely to file complaints than emergency patients.^[15]^

Emergency reconstruction of thumb amputation did not increase the complications of patients, and emergency reconstruction was safe. Emergency reconstruction can maximize the use of valuable blood vessels, nerves, tendons, and bone and joint tissues. Emergency reconstruction of the thumb can reduce operation time and hospitalization time, reduce operation costs, and obtain a more ideal appearance and function. However, due to the complexity of thumb reconstruction surgery, there are few clinical cases, which leads to certain limitations in the study. In the future, we will conduct more comparative studies to increase the reliability of the results.

In conclusion, there is no difference in the treatment effect between emergency and elective surgery for thumb reconstruction. Emergency surgery can reduce the length of hospital stay, promote patient recovery, and improve patient satisfaction. Therefore, we advocate early emergency reconstruction.

## Author contributions

Conceptualization: Lei Ge, Yang Gao, Qiandong Liu

Data curation: Lei Ge, Xiangyun Wang, Qiang He

Formal analysis: Lei Ge, Lei Zhang

Investigation: Yang Gao, Libin Lu

Resources: Qiandong Liu, Qinglin Dong

Software: Qiang He, Lei Zhang, Libin Lu

Writing – original draft: Lei Ge, Qiandong Liu

Writing – review & editing: Qiandong Liu.
